# Role for 2D image generated 3D face models in the rehabilitation of facial palsy

**DOI:** 10.1049/htl.2017.0023

**Published:** 2017-08-10

**Authors:** Gary Storey, Richard Jiang, Ahmed Bouridane

**Affiliations:** Department of Computer and Information Sciences, Northumbria University, Newcastle Upon-Tyne NE21XE, UK

**Keywords:** patient rehabilitation, computer vision, medical image processing, stereo image processing, 2D image generated 3D face models, facial palsy rehabilitation, computer vision, hybrid 2D facial landmark htting technique

## Abstract

The outcome for patients diagnosed with facial palsy has been shown to be linked to rehabilitation. Dense 3D morphable models have been shown within the computer vision to create accurate representations of human faces even from single 2D images. This has the potential to provide feedback to both the patient and medical expert dealing with the rehabilitation plan. It is proposed that a framework for the creation and measuring of patient facial movement consisting of a hybrid 2D facial landmark fitting technique which shows better accuracy in testing than current methods and 3D model fitting.

## 1 Introduction

Recent medical studies [1–3] have highlighted that patients diagnosed and treated with specific types of facial paralysis such as Bell's palsy have outcomes that are directly linked to the rehabilitation provided. While various treatment and rehabilitation paths exist dependant on the specifics of the facial palsy diagnosis, the aim is to restore a degree of facial muscle movement to the patient. Lindsay *et al* [4] completed a comprehensive study over 5 years of the rehabilitation process and outcomes for 303 facial paralysis patients, the key finding was the need for specialised therapy plans tailored via feedback for the best patient outcomes. While Banks *et al* [5] have shown that quality qualitative feedback to a clinician is required for the best development of rehabilitation plans.

Tracking and providing qualitative feedback on the progress of rehabilitation for a patient is an area where the application of computer vision and machine learning techniques could prove to be highly beneficial. Computer vision methods can provide the capability of capturing accurate 3D models of the human face these in turn can be leveraged to analyse and measure changes in face shape and levels of motion [6].

Applying 3D face modelling techniques in an automated framework for tracking facial palsy rehabilitation progression has a number of potential benefits. 3D face models generated from a 2D face image can provide a detailed topography of an individual human face which can be qualitatively measured for change over time by a computer system. Potential benefits of such an automated system include providing the clinician dealing with a patients rehabilitation to gather regular objective feedback on the condition and tailor therapy without always needing to physically see the patient or providing continuity of care if for instance the clinician changes during the rehabilitation period. Patients will have a visual evidence in which to see the progress that has been made. It has been indicated that patients suffering from facial palsy can also be affected by psychological and social problems the capacity to track rehabilitation privately within a comfortable setting like their own home may be of benefit.

Some previous studies [7] have looked at the process of aiding diagnosis through the application of computer vision techniques these have been limited to 2D imaging which measure on a spare set of landmarks. The hypothesis is that 3D face modelling consisting of thousands of landmarks provides a far richer model of the face which in turn can present a more accurate measurement system for facial motion.

In this Letter we propose a framework applicable for accurate generation of 3D face models of facial palsy patients from 2D images applying state-of-the-art methods and a proposed method of using geometrical features to track rehabilitation and present our conclusions.

## 2 Proposed system overview

The accuracy of the facial representation is a key components of any computer-based system which aims to measure facial motion. We suggest that the more complex a depiction of the individuals patient facial topography the greater the potential is for the desired level of accuracy. Developing such a system requires a framework of methods to build and measure such a model.

**Fig. 1:**
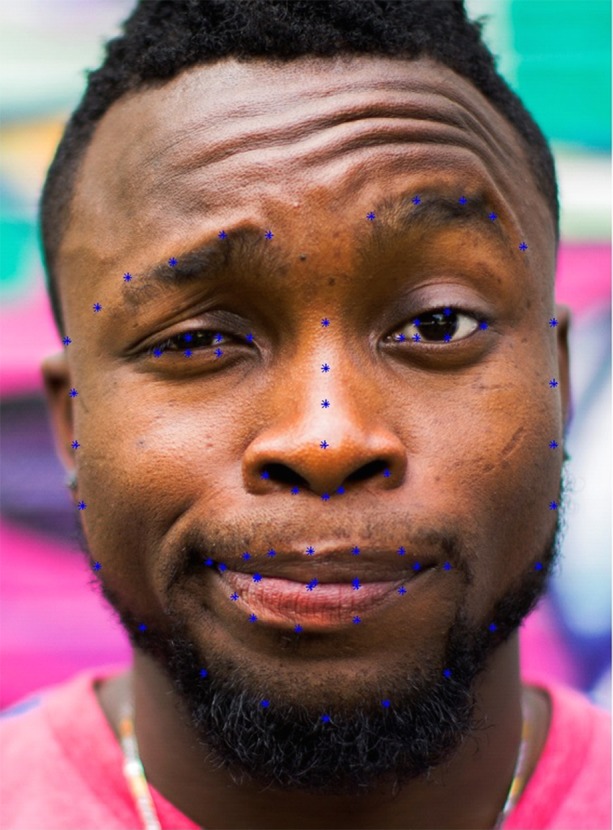
2D face alignment of 68 landmarks on a facial image which displays asymmetric movement, like that of a patient suffering from facial palsy

As camera systems which perceive depth within an image are not currently common place or require specialist and expensive hardware initially we require a method for face detection and 2D face alignment. Fig. 1 shows an example of 2D face alignment where 68 landmark fitted to the face. Many methods have been researched for this purpose and in the limited previous work on facial palsy the method have adopted a variation of the active shape model [7]. Over the recent other method have shown state-of-the-art results such as discriminative response map fitting (DRMF) [8], deformable part models (DPM) [9] and more recently a deep learning variation which applies convolutional neural (CNN) networks for pose-invariant 3D face alignment (PIFA) [10].

**Fig. 2:**
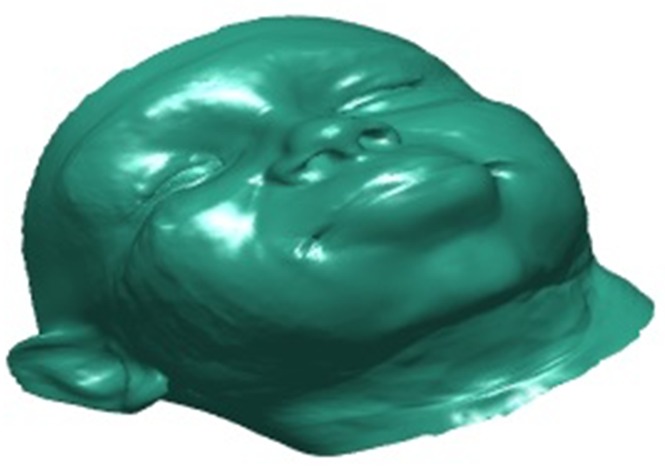
An example of a 3D morpahable model, fitted to the facial image shown within Fig. 1

Following the 2D face alignment process we propose the generation of a 3D facial model. 3D facial modelling provides a much richer representation of a individuals facial geometry comprised of a dense mesh generally contains many thousand vertices'. The use of 3D face models theoretically provides us with a set of geometric features which can provide a more accurate measure of facial movements in our prosed system the 3D morphable model (3DMM) [6] is applied. 3DMM have been shown to produce accurate models in research and recent 3DMM fitting approaches in [11] has shown excellent results as demonstrated by the fitted model shown in Fig. 2.

**Fig. 3:**
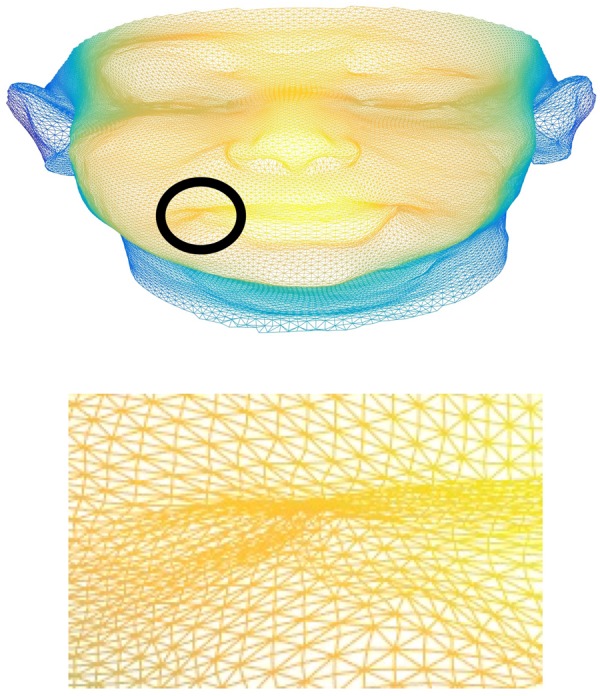
Example of a 3D face models dense mesh of vertices' that describe the face geometry

Once a 3D face model is generated a set of features is required to be used for measuring the facial motion. Geometric features have previously been shown to be in areas such as facial expression recognition [12] which shares some similarities with this problem domain. With a larger set of key-points (example of which is shown in Fig. 3) that describe the face in rich detail we believe that geometric feature have the potential to measure facial movement ranges with a greater degree of accuracy. Extraction of a feature set based upon geometric features is also relatively computationally inexpensive.

## 3 Methods

Our framework consists of three specific components which are 2D face alignment, 3DMM fitting and geometric feature extraction. Within this section each components methods are discussed in further detail.

To provide the most accurate detection of 2D facial landmarks we propose a hybrid method based upon our experimental findings discussed later in this Letter. This hybrid method consists of applying two distinct methods each fitting a subset of the 2D facial landmarks. The first 2D facial alignment method for fitting a majority of the landmarks required to construct a 3DMM is DRMF, which is a form of a parts based constrained local models (CLM). The model is setup as $M = \lcub S\comma \; D\rcub $ in which a set of detectors *D* of the various facial landmarks corresponds to fiducial points of the shape model *S*. CLMs define a face as a 3D object as follows

(1) $$s\lpar p\rpar = sR\lpar s_0 + \Phi _sq\rpar + {\bi t}\eqno\lpar 1\rpar $$

In (1) *R* is a rotation component where $R = \lsqb r_x\semicolon \; r_y\semicolon \; r_z\rsqb $, *s* define scale and ${\bi t}$ is the translation vector as ${\bi t} = \lsqb t_x\semicolon \; t_y\semicolon \; 0\rsqb $. Non-rigid variations of the shape are controlled by *q*. The parameters of the shape model are therefore $p = \lsqb s\comma \; r_x\comma \; r_y\comma \; r_z\comma \; t_x\comma \; t_y\comma \; q\rsqb $. The detectors *D* are a set of linear classifiers which detect *n* parts of the face as $D = \lcub w_i\comma \; b_i\rcub _{i = 1}^n $. A linear detector for the $i{\rm th}$ part of the face such as the chin are $w_i$ and $b_i$ which are applied to define probability maps for the $i{\rm th}$ part given a location *x* given an face image *I* as

(2) $$p\lpar l_i = 1\vert x\comma \; I\rpar = \displaystyle{1 \over {1 + {\rm e}^{\lcub l_i\lpar w_i^{\rm T} f\lpar x\comma I\rpar + b_i\rpar \rcub }}}\eqno\lpar 2\rpar $$

In (2) $f\lpar x\semicolon \; I\rpar $ is the feature extracted from the patch in image *I* centred at $x_i$. The probability of not being correctly spotted at *x* is $p\lpar l_i = - 1\vert x\comma \; I\rpar = 1 - p\lpar l_i = 1\vert x\comma \; I\rpar $. The DRMF method applies a discriminative regression framework for estimating the model parameters *p*. Specifically it introduces a perturbation $\Delta p$ and around each point of the perturbed shape are response estimates in a $w \times w$ window which is centred around the perturbed point, $A_i\lpar \Delta p\rpar = \lsqb p\lpar l_i = 1\vert x + x_i\lpar \Delta p\rpar \rsqb $. From the response maps around the perturbed shape $\lcub A_i\lpar \Delta p\rpar \rcub _{i = 1}^n $, a function *f* is learnt such that $f\lpar \lcub A_i\lpar \Delta p\rpar \rcub _{i = 1}^n = \Delta p$. For brevity we refer the reader to [8] for a full technical overview of the DRMF method.

The second method applied for fitting the important mouth region landmarks we apply the PIFA method [10]. PIFA applies a series of CNNs within a cascaded regression framework is to estimate the shape parameter *p*. A mapping to predict *p* is learnt from a $N_d$ set of training images. An estimated update to the shape parameter at the $k{\rm th}$ stage of the cascaded CNN is learnt as per eqn. (3) where the true shape update is the difference between the current shape parameter and the ground truth as $\Delta p_i^k = p_i^k - p_i^{k - 1} $, $I_i$ is the training image, $U_i$ is current estimated 2D landmarks and $v_i^{k - 1} $ is estimated landmark visibility

(3) $$\Theta _k^p = \mathop {{\rm arg}\; {\rm min}}\limits_{\Theta _k^p } \sum\limits_{i = 1}^{N_d} \left\Vert {\Delta p_i^k - {\rm CNN}_p^k \lpar I_i\comma \; U_i\comma \; v_i^k \comma \; \Theta _k^p \rpar } \right\Vert ^2\eqno\lpar 3\rpar $$

A six-stage cascaded CNN is used, at the initial input stage CNN $_m^1 $ the entire face region scaled to 114 × 114 is used, then at subsequent stage a 114 × 114 image containing an array of 19 × 19 pose-invariant feature patches, extracted from the current estimated 2D landmarks. We refer the reader to [10] for a more comprehensive overview of the method and the novel feature patches employed.

We concatenate the set of 2D facial landmarks from the relevant points of the outcomes from the DRMF and PIFA methods, these are passed to the second stage of the framework in which a 3DMM is generated. The 3DMM is used to represent a dense 3D shape of an individual's face in our framework we apply the fitting technique as described by [13]

(4) $$S = \bar S + A_{id}\alpha _{id} + A_{{\rm exp}}\alpha _{{\rm exp}}\eqno\lpar 4\rpar $$

*S* describes the 3D face where $\bar S$ is the mean shape, $A_{id}$ and $A_{{\rm exp}}$ are the principle axes trained on the 3D face scans with neutral expression and expression scans, respectively. While $\alpha _{id}$ are the shape parameters and $\alpha _{{\rm exp}}$ the expression parameters. $A_{id}$ and $A_{{\rm exp}}$ are provided by the Basel Face Model [14] and Face-Warehouse [15], respectively.

A weak perspective projection is used to project the face model to the image plain for the fitting of the 3DMM to a face image

(5) $$s_{2d} = fPR\lpar S + t_{3d}\rpar \eqno\lpar 5\rpar $$

$s_{2d}$ are the 2D positions of 3D points on the image plane, *f* denotes the scaling factor, *P* is the orthographic projection matrix $\left({\matrix{ 1 & 0 & 0 \cr 0 & 1 & 0 \cr } } \right)$, $R = \lpar \alpha \comma \; \beta \comma \; \gamma \rpar $ is the 3 × 3 rotation matrix constructed with pitch$\lpar \alpha \rpar $, yaw$\lpar \beta \rpar $ and roll$\lpar \gamma \rpar $ and $t_{3d}$ is the translation vector.

The fitting of this model is defined by (6) where the 2D landmarks identified in stage one of the framework defined here as $s_{2{\rm d}t}$ associated 3D points and estimate the model parameters by minimising the distance between $s_{2d}$ and $s_{2{\rm d}t}$.

(6) $$\mathop {{\rm arg}\, {\rm min}}\limits_{_{\,f\comma R\comma t_{3d}\comma \alpha _{id}\comma \alpha _{{\rm exp}}} } \left\Vert {s_{2{\rm d}t} - s_{2d}} \right\Vert \eqno\lpar 6\rpar $$

A fitted 3D face model *S* is a dense mesh consisting of *m* vertices where $m = 53215$ in the example shown in Fig. 3.

We propose an initial technique for extracting *n* relevant geometric feature sets that can be applied to measure and track the restoration of facial motion

(7) $$E_i = \lpar S\lpar \colon \comma \; d\rpar _1 - S\lpar \colon \comma \; d\rpar _0\rpar ^2\eqno\lpar 7\rpar $$

In (7) a set of evaluations *E* are defined by the clinician which forms the basis for measuring the rehabilitation progress of the patient. $S_0$ defines the 3D face model at a neutral expression, while $S_1$ is the model at end range of the prescribed evaluation movement. *d* defines a *N*-dim index vector indicating the indexes of semantically meaningful 3D vertexes. As facial palsy often affects the facial movement in an asymmetrical manner between the left and right side while also the range of affected musculature is not always equal between the upper (eye and brow region) and lower (mouth region), the semantically meaningful 3D vertexes will differ for patients though are likely to be quadrant or region based

(8) $$R\lpar E_1\comma \; E_{1 + i}\rpar = E_{1 + i} - E_1\eqno\lpar 8\rpar $$

Equation (8) defines a basic rehabilitation measurement where $E_1$ is the set of initial evaluation taken pre-rehabilitation and $E_{1 + i}$ is the most recent set of evaluations. A more semantically meaningful metric could be provided through the incorporation of a mapping to one of the recognised medical grading systems for facial palsy such Yanagihara, House–Brackmann or Sunnybrook.

## 4 Results

A private data set of six individuals who have a confirmed diagnosis of facial palsy are used to conduct some initial tests on the capability of the prosed hybrid method for fitting 2D facial landmarks. Each image is a cropped full frontal facial images which have been manually marked with a 68 facial landmarks to be used as the ground truth landmark positions. For testing purposes a subset of landmarks are applied that are identically marked for each of the methods tested. The methods test are DRMF [8], PIFA [10], DPM [9] where we apply both a fully independent part model and also a shared part model using 99 parts released by the authors. Finally we show results for the mention hybrid approach where PIFA is used to fit the landmarks relating to the mouth and DRMF for the other landmarks. We apply the root mean square error with ocular scaling to deal with the images having different size faces as used in [8] to measure the accuracy of the techniques.

**Fig. 4:**
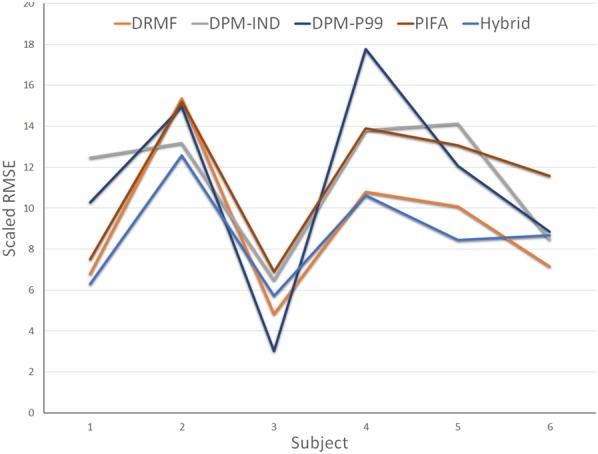
Results showing root mean square error per subject in the dataset

Fig. 4 shows that across the dataset the accuracy of methods on a subject to subject basis can vary to a fairly large margin. DPM shared model is especially volatile giving the best fit for subject 3 but by far the worst for subject 4. While not performing the best for any subject the proposed hybrid method is the consistent in terms of accuracy across the dataset. When examining the mean RMSE as shown in Table 1 we can see that the hybrid performs above all other methods with DRMF also showing a distinct advantage over the other methods including the state-of-the-art CNN-based PIFA method.

**Table 1:** Total root mean square error per method

Method	Total RMSE
DRMF	9.17
DPM independent	11.42
DPM 99 part shared	11.16
PIFA	11.36
hybrid	8.72

**Fig. 5:**
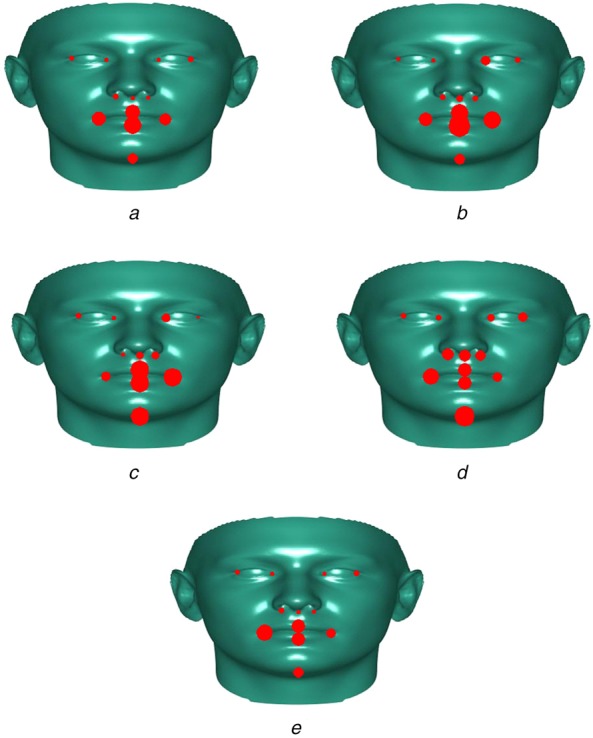
Results showing root mean square error per landmark with larger landmarks representing a larger error *A* DRMF *B* DPM independent *C* DPM 99 Part Shared *D* PIFA *E* Hybrid

When we further examine the results as shown in Fig. 5 based on the accuracy for certain key facial landmarks DRMF has very high accuracy for all landmarks except for the mouth area. Whereas the PIFA method fits mouth landmarks better but struggles on this dataset with accuracy in other areas. The hybrid method provides the best accuracy though there is still some issues when fitting the corners of the mouth. This is likely due to the asymmetrical nature of the mouth location on the test set and that none of the models tested have been trained on any data specifically relating to this condition.

## 5 Conclusions

In this Letter we have proposed a potential framework for measuring the progress of rehabilitation for patients with facial palsy through automatically building a 3D face model from basic 2D images of the patient. We have investigated landmark fitting methods using state-of-the-art techniques and proposed a hybrid 2D landmark fitting method incorporating these which provides better accuracy when measured against the ground truth 2D images.

To realise the potential of an application for facial palsy rehabilitation measurement there two key areas of further work. The first is that although the hybrid method proposed provides a high degree of accuracy on landmark fitting a significant level of error resides in fitting mouth landmarks specifically in facial palsy patients when there is a large range of asynchronous movement. This level of error could negatively impact the accuracy of the rehabilitation tracking and therefore further study of asymmetrical motion in the face needs to be captured with 2D landmark fitting systems. The second is to develop available datasets of facial palsy specifically graded 3D models which can be used as ground truth to fully support the proposed framework in its entirety.
